# *Neisseria gonorrhoeae* non-susceptible to cephalosporins and quinolones in Northwest Ethiopia

**DOI:** 10.1186/1471-2334-13-415

**Published:** 2013-09-05

**Authors:** Martha Tibebu, Ambachew Shibabaw, Girmay Medhin, Afework Kassu

**Affiliations:** 1 Department of Medical Microbiology, Immunology and Parasitology, Bahir Dar University, Bahir Dar, Ethiopia; 2 Amhara Regional Health Research Laboratory Bahir Dar Center, P. O. Box 641, Bahir Dar, Ethiopia; 3 Department of Epidemiology and Biostatistics, Institute of Pathobiology, Addis Ababa University, P. O. Box 1176, Addis Ababa, Ethiopia; 4 Department of Microbiology, School of Biomedical and Laboratory Sciences, College of Medicine and Health Sciences, University of Gondar, P.O. Box 196, Gondar, Ethiopia

**Keywords:** Non-susceptible, *Neisseria gonorrhoeae*, Ethiopia

## Abstract

**Background:**

The occurrence of antibiotic resistant *Neisseria gonorrhoeae* isolates is a serious public health problem in different corners of the globe. The objective of this study was to analyze the antimicrobial susceptibility pattern of *N. gonorrhoeae* in Northwest Ethiopia.

**Methods:**

This was a retrospective study of *N. gonorrhoeae* isolated from genital swabs of patients referred to the Amhara Regional Health Research Laboratory between September 2006 and June 2012 in Bahir Dar, Ethiopia. A structured check list was used to collect socio-demographic and laboratory variables. Data were analyzed using SPSS software version 16.

**Results:**

Out of 352 genital specimens processed, 29 clinical strains of *N. gonorrhoeae* were identified. The percentage of *N. gonorrhoeae* isolates non-susceptible to ceftriaxone, ciprofloxacin, tetracycline and penicillin G was 27.8%, 40.9%, 92.6% and 94.4% respectively. Twenty percent of the isolates were found to be non-susceptible to both ceftriaxone and ciprofloxacin. Non-susceptibility to an injectable cephalosporin and any two of quinolones, penicillins or tetracyclines was observed in 27.8% of the isolates. The percentage of *N. gonorrhoeae* which were non-susceptible to tetracycline or penicillin G was high throughout the study period. However, the percentage of fluoroquinolone or cephalosporine non-susceptible strains showed an increasing trend.

**Conclusions:**

A high percentage of *N. gonorrhoeae* isolated from genital specimens in Northwest Ethiopia are non-susceptible to an injectable cephalosporin and any two of quinolones, penicillins or tetracyclines. Treatment of gonorrhea in the study area needs to be guided by antibiotic susceptibility testing of isolates.

## Background

Gonococcal infections are the second most common prevalent sexually transmitted bacterial infections causing substantial morbidity worldwide each year [1]. In addition to causing serious complications, gonorrhea is a potent amplifier of the spread of sexually transmitted HIV [2] increasing the infective inoculums of HIV in persons with HIV and gonorrhea co-infection.

Resistance to commonly-prescribed antibiotics of *Neisseria gonorrhoeae* is an expanding global problem resulting in diminishing treatment options for gonorrhea. Loss of utility of several drugs such as sulfonamides, penicillins and tetracyclines, for treatment of gonorrhea, were reported in both developed and developing countries [3,4].

Until recently, quinolones and third-generation cephalosporins had activity against *N. gonorrhoeae* strains that were resistant to previously used antibiotics such as sulfonamides or tetracyclines. However, the emergence and spread of gonococci resistant to the quinolone group of antibacterials was reported from different corners of the world [5,6]. Failures of treatment with oral third-generation cephalosporins for infection with gonococci or raised minimum inhibitory concentrations to them have been documented in different continents including Europe, Asia and North America [7-10].

Although individual country-based intermittent analyses about drug resistant gonococci have been published from some countries, information about the exact magnitude of multidrug resistant (MDR) *N. gonorrhoeae* is generally lacking for most of African countries including Ethiopia. The current treatment guidelines in Ethiopia, and some other African countries, suggest the syndromic case management for all sexually transmitted infections (STIs) including gonorrhea based on the WHO recommendation [11].

With the occurrence of resistance to commonly prescribed antibiotics in both developed and developing countries, an updated knowledge of the prevailing susceptibility patterns of gonococci in Ethiopia is important for the proper selection and use of antimicrobial drugs as well as for the development of an appropriate prescription policy. This study was aimed to investigate the antibiotic susceptibility patterns of *N. gonorrhoeae* isolated from genital swab of patients seen in the Amhara Regional Health Research Laboratory (ARHRL), Bahir Dar, Ethiopia.

## Methods

### Study design and period

In this retrospective study, genital specimen analysis data from bacteriology laboratory registers, which were documented between September 2006 and June 2012 in the ARHRL, Bahir Dar Center, Ethiopia was used.

### Study area

The ARHRL Bahir Dar Center is the main referral laboratory in the Amhara National Regional State (ANRS). The laboratory is located in Bahir Dar, the capital of the ANRS. Patients get referred to this laboratory from both private and government owned health facilities located in the administrative divisions in the ANRS as well as from adjacent regions in the country.

### Source population, study participants

The source populations were patients in the ANRS who were referred to the ARHL Bahir Dar Center for laboratory work up. All genital specimen analyses reports between September 2006 and June 2012 were included in this study.

### Laboratory methods

As part of the routine bacteriologic investigations at ARHRL, all genital specimens were plated onto modified Thayer Martin media immediately after collection in the laboratory and incubated at 37°C in 5% CO_2_. The plates were read daily for up to 72 hours and isolates were identified by conventional phenotypic and biochemical methods. Gram negative diplococcic which were oxidase and catalase positive were considered as *N. gonorrhoeae.* Susceptibility testing was performed by Kirby-Bauer disc diffusion method using penicillin G (10 ug), tetracycline (30 ug), ciprofloxacin (5 ug) and ceftriaxone (30 ug). CLSI guidelines were used for interpretation of zones of inhibition and quality control.

### Data extraction methods

A structured check list was used to collect socio-demographic and laboratory data such as the antibiotic susceptibility test results and year of test. The laboratory records did not specify the type of symptoms or any other clinical information.

In order to analyze trends of antibiotic resistance by *N. gonorrhoeae*, the study period was divided into three categories, i.e., September 2006 – August 2009, September 2009 – August 2011 and September 2011 - June 2012.

### Data quality management, processing and analysis

Data were double-entered using Epi info version 3.5 and analyzed using SPSS software version 16.

### Operational definitions

Non-susceptible *N. gonorrhoeae* isolates were defined as those that are not sensitive to the antibiotic tested for susceptibility, i.e., those isolates exhibiting resistance or intermediate resistance.

Dual non-susceptibility: non-susceptibility to any two of the antibiotics tested for susceptibility.

Multi Drug Non-susceptibility: Combined non-susceptibility to an injectable cephalosporin and any two of quinolones, penicillins or tetracyclines.

### Ethical considerations

Ethical approval to conduct the study was obtained from the research and technology transfer core process of the ANRH bureau. Permission to conduct the study was granted from the ARHRL, Bahir Dar center.

## Results

Between September 2006 and June 2012, 352 genital specimens were analyzed in ARHRL, Bahir Dar Center by bacterial culture following the standard operating procedures. The mean age of the participants was 28.1 years (SD = 8.4), with female to male ratio of 1.1. As indicated on Table 1, there were 29 clinical strains of *N. gonorrhoeae* isolated and processed for susceptibility testing during the study period.

**Table 1:** **Number of specimens and *N. gonorrhoeae *isolated from September 2006to June 2012, ANRS, Ethiopia**

**Year category (duration)**	**Total number of genital specimen processed**	**Total number of *N. gonorrhoeae *isolated**	**Period prevalence (95%CI) of *N. gonorrhoeae *isolated**
Sept. 2006 to Aug. 2009 (36 months)	111	9	8.1% (3.7%, 14.8%)
Sept. 2009 to Aug. 2011 (24 months)	119	7	5.9% (2.4%, 11.7%)
Sept. 2011 to June 2012 (10 months)	122	13	10.7% (5.8%, 17.5%)
Total	352	29	8.2% (5.6%, 11.6%)

Table 2 shows the antibiotic susceptibility pattern of the *N. gonorrhoeae* isolates. In toto, as much as 27.8% of the isolates were non-susceptible to ceftriaxone. Non-susceptibility to ciprofloxacin was noticed in 40.9% of the isolates. High percentage of non-susceptibility was observed against penicillin G (94.4%) and tetracycline (92.6%). Non-susceptibility to an injectable cephalosporin coupled to any two of penicillins, ciprofloxacin or tetracycline was identified in 27.8% of the isolates. Dual non-susceptibility to ceftriaxone and ciprofloxacin was noticed in 20% of the isolates, all of which were documented between Sept. 2011 and June 2012.

**Table 2:** **Antibiotic susceptibility profile of *N. gonorrhoeae *isolates from September 2006to June 2012, ANRS, Ethiopia**

**Antimicrobial agent**	**Percent non-susceptibility**	**95% confidence intervals**
**Ceftriaxone**	27.8	(9.7, 53.5)
**Penicillin G**	94.4	(72.7, 99.9)
**Tetracycline**	92.6	(75.7, 99.1)
**Ciprofloxacin**	40.9	(20.7, 63.6)
**Ceftriaxone and Penicillin G**	35.7	(12.8, 64.9)
**Ceftriaxone and Tetracycline**	31.3	(11.1, 58.7)
**Ceftriaxone and Ciprofloxacin**	20	(4.3, 48.1)
**Penicillin G and Tetracycline**	87.5	(61.7, 98.4)
**Penicillin G and Ciprofloxacin**	66.7	(34.9, 90.1)
**Tetracycline and Ciprofloxacin**	38.1	(18.1, 61.6)
**Multi drug non-susceptibility**	27.8	(9.7, 53.5)
**Other triple non-susceptibility**	63.6	(30.8, 89.1)
**All four drugs non-susceptibility**	27.3	(6.0, 61.0)

The percentage of isolates non-susceptible to ciprofloxacin recorded between September 2009 to August 2011 was 11.1%. Between September 2011 to June 2012, the percentage of non-susceptibility documented against ciprofloxacin was 77.8%. Non-susceptibility to ceftriaxone was demonstrated by 20% of the isolates between September 2006 and August 2009 while it was 80% between September 2011 to June 2012. The percentage of *N. gonorrhoeae* isolates which were non-susceptible to tetracycline or penicillin G was high over the past 6 years. However, percentage non-susceptibility to fluoroquinolones or cephalosporins showed an increasing trend during the study period, as indicated on Table 3.

**Table 3:** **Trends in antibiotic non-susceptibility of clinical *N. gonrrhoeae *isolates in the Amhara regional health research laboratory center between September 2006and August 2009**

**Antimicrobial agent**	**September 2006 to August 2007**	**September 2007 to August 2008**	**September 2008 to August 2009**
**Ceftriaxone**	20 (1/5)	0	80 (4/5)
**Penicillin G**	23.5 (4/17)	17.7 (3/17)	58.8 (10/17)
**Tetracycline**	28.0 (7/25)	24.0 (6/25)	48.0 (12/25)
**Ciprofloxacin**	11.1 (1/9)	11.1 (1/9)	77.8 (7/9)
**Ceftriaxone and Penicillin G**	20 (1/5)	0	80 (4/5)
**Ceftriaxone and Tetracycline**	20 (1/5)	0	80 (4/5)
**Ceftriaxone and Ciprofloxacin**	0	0	100 (3/3)
**Penicillin G and Tetracycline**	21.4 (3/14)	14.3 (2/14)	64.3 (9/14)
**Penicillin G and Ciprofloxacin**	0	12.5 (1/8)	87.5 (7/8)
**Tetracycline and Ciprofloxacin**	12.5 (1/8)	0	87.5 (7/8)
**Multi drug non-susceptibility**	20 (1/5)	0	80 (4/5)

Numbers in each cell in the body of the table are: Percent (Number of non-susceptible isolate/Total isolates tested for the antimicrobial agent in that year band).

## Discussion

For the first time, this study reported an evidence of reduced susceptibility to common and new generation antibiotics of *N. gonorrhoeae* isolates in Northwest Ethiopia. It also revealed increasing trend in percentages of cephalosporin or fluoroquinolone non-susceptible isolates while that of old, inexpensive antibiotics such as penicillin and tetracycline was maintained at a high level.

Although the main stay of management of discharge syndromes in the study area is the syndromic approach, the number of isolates being sent for bacteriological analysis showed an increasing tendency with time. As opposed to the etiological approach which uses laboratory tests to identify the causative agents of STIs, the syndromic approach leads to immediate treatment for possible causes of one of seven STI syndromes based on symptoms only [12]. This indicates a gradual shift to the etiologic approach of diagnosis of gonorrhea. The occurrence of *N. gonorrhoeae* isolates non-susceptible to the available antibiotics could be the underlying factor to the increased request for the exact identification of etiologic agents and determination of their susceptibility patterns.

In this study, a high level of non-susceptibility to new generation antibiotics such as ciprofloxacin and ceftriaxone was noticed. The high proportion of fluoroquinolone non-susceptibility by *N. gonorrhoeae* is coherent with reports from many countries including South Africa, Kenya and the Southeast Asia region [13-15]. The reason for this consistency may be the global nature of gonorrhea. Those infected in one part of the world will often spread the infection to those living elsewhere if ineffective treatment is used. However, the roles of spontaneous mutation and the abilities of bacteria to adapt to different environmental situations to the occurrence of MDR *N. gonorrhoeae* in the study area cannot be ruled out.

However, low levels of non-susceptibility to ciprofloxacin were reported from a multi-centered study conducted in Central African Republic, Cameroon and Madagascar [16]. The inconsistency of our finding with these reports may be the difference in time frame between the studies.

With the introduction of syndromic management for STIs [12], the use of fluoroquinolones and cephalosporins is practiced in a standardized fashion. However, this creates a selective advantage for the organisms resistant to these medications [17]. As a result, *N. gonorrhoeae* having reduced susceptibility to such drugs would occur. Use of fluoroquinolones and cephalosporins for treatment of other conditions may lead to sensitization of the *N. gonorrhoeae* isolates in asymptomatic patients with gradual selection of drug resistant strains and eventually clinically significant resistance.

The high levels of tetracycline and penicillin G non-susceptibility reported in this study parallel earlier experience of other countries such as Malawi and Kissumu, Kenya [3,4]. As Gedebou M and Tasew A had reported the presence of a relatively lower level of resistance of *N. gonorrhoeae* to tetracycline (8%) and Penicillin (41%) in Ethiopia three decades ago [18], the current magnitude of reduced susceptibility to these drugs seems to have developed gradually. Also a study to determine the susceptibility patterns of *N. gonorrhoeae* isolates in Northwest Ethiopia had reported, in 2001, lower resistance rates to penicillin G (85.2%), tetracycline (29.6%) and cefrtriaxone (4.2%) [19], thus supporting the gradual nature of the condition.

The finding of the presence of *N. gonorrhoeae* having reduced susceptibility to an injectable cephalosporine and any two of quinolones, penicillins or tetracyclines in the study area is coherent with the reports from other areas such as the Western Pacific region [20]. Based on the WHO recommendation that an antimicrobial associated with a resistance of  ≥ 5% of strains should be abandoned [11], the use of cephalosporins, ciprofloxacin, penicillin G or tetracyclines for the treatment of gonorrhea in the general population in the ANRS would be questionable.

Currently, the Centers for Disease Control and Prevention (CDC) recommends dual therapy with ceftriaxone (250 mg intramuscularly as a single dose plus either azithromycin 1 gram orally as a single dose or doxycycline 100 mg orally twice a day for 7 days as the most effective treatment for uncomplicated gonorrhea [21]. Even though it is not possible to have data about the susceptibility patterns of *N. gonorrhoeae* isolates for doxycycline in the study area, it is theoretically known that microorganisms that have become insensitive to one tetracycline invariably exhibit cross resistance to other tetracyclines due to transmissible plasmids [22]. Therefore, based on the finding that there is high proportion of ceftriaxone-tetracycline dual non-susceptibility, the use of ceftriaxone-doxycycline combination for the treatment of gonorrhea in the study area needs caution.

The results from this study are alarming because this level of non-susceptibility to common and new generation antibiotics is being reported in a setting where there are only few alternatives for the management of gonorrhea. Since antibiotic treatment is the foundation of gonorrhea management, the emergence of *N. gonorrhoeae* having reduced susceptibility to the available and affordable antibiotics can be associated with an increase in the risk of complications of infection, hence a challenge to clinicians. The occurrence of untreatable gonorrhea in a country that already has diseases such as tuberculosis, HIV and malaria [23] as the public health priorities is an additional burden. It would threaten effective disease control because there are no effective antimicrobial resistance surveillance programs for *N. gonorrhoeae* in the ANRS as well as in other parts of Ethiopia. The impact on HIV transmission could also be very serious because in the ANRS the prevalence of HIV is high [24].

The findings in this report are subject to the following limitations. First, the data presented here are based on results reported on laboratory registers; due to the retrospective nature of the study, documentation problems are an issue. Second, susceptibility testing was done by the Kirby-Bauer disc susceptibility testing method, which is unsuitable for the laboratory differentiation of partially resistant from fully resistant strains of *N. gonorrhoeae*[25], thus necessitating the strict use of the word ‘non-susceptible’ instead of ‘resistant’. Third, data available only include results from genital gonococcal isolates among patients who were referred for laboratory analysis. As patients who respond to empiric treatment are not sent for laboratory analysis, subjects included in this study are those who were already exposed for different antibiotics. That individuals at risk for the occurrence of antibiotic resistance are the study subjects makes selection bias unavoidable.

The other is the small number of isolates documented and analyzed over the years. This was mainly related to the policy in Ethiopia which recommends syndromic case management of STI cases as opposed to the etiologic approach which would have lead to laboratory analysis of more isolates and determination of drug susceptibility patterns. However, in light of similar trends in other regions of the world, the patterns observed in this study coupled to the ability of *N. gonorrhoeae* to develop resistance are concerning enough.

## Conclusions

High proportion of *N. gonorrhoeae* isolated from genital specimens in the ANRS are non-susceptible to old and new generation antibiotics including to a combination of an injectable cephalosporin and any two of quinolones, penicillins or tetracyclines. In view of this, the national guideline for management of gonorrhea needs to be urgently reviewed. The diagnostic approach needs to be modified to include determination of susceptibility patterns of *N. gonorrhoeae* isolates. Clinicians need to consider treatment of gonorrhea based on individual susceptibility patterns instead of the routine empirical treatment of discharge syndromes. A strategy should be established to rapidly detect patients diagnosed with gonorrhea who experience a clinical treatment failure following treatment with the recommended antibiotics. Current treatment recommendations for gonorrhea in the study area need to be validated and updated. Protocols for management of gonorrhea treatment failures need to be put in place. Antimicrobial resistance surveillance program needs to be launched in ANRS in order to obtain antimicrobial resistance profiles in a timely manner and with sufficient clinical and epidemiological information. Further studies with determination of MIC need to be conducted in the study area in order to determine the exact level of bacterial resistance.

## Competing interests

The authors declare that they have no competing interests.

## Authors’ contributions

MT wrote the proposal, analyzed the data and drafted the paper. AS participated in data collection, entry and analysis. GM and AK participated in the analysis and reviewed the manuscript. All authors participated in the preparation of the manuscript and approved the final manuscript.

## Pre-publication history

The pre-publication history for this paper can be accessed here:

http://www.biomedcentral.com/1471-2334/13/415/prepub
